# P13 Calciphylaxis as a rare skin manifestation in a patient with an inflammatory myositis

**DOI:** 10.1093/rap/rkad070.034

**Published:** 2023-09-27

**Authors:** Syeda Tabassum, Kirsty Levasseur

**Affiliations:** The Dudley Group NHS Foundation Trust, Dudley, United Kingdom; The Dudley Group NHS Foundation Group, Dudley, United Kingdom

## Abstract

**Introduction:**

Calciphylaxis is a rare but severe vascular complication that can occur in patients with inflammatory myositis. It is characterised by an abnormal deposition of calcium in small and medium sized vessels which can then lead to occlusion and tissue ischemia. It is also known as calcific uremic arteriolopathy, primarily associated with end-stage renal disease, but can also manifest in other clinical settings, including myositis. Myositis encompasses various autoimmune disorders such as polymyositis and dermatomyositis. The exact mechanisms underlying the development of calciphylaxis in myositis are not entirely understood. However, chronic inflammation associated with myositis can trigger this condition.

**Case description:**

A 70-year-old woman presented to the emergency department in April 2021 with one month history of persistent shortness of breath on exertion and fatigue. A thorough investigation was carried out with auto-immune profile being negative and the radiological appearances were consistent with cryptogenic organizing pneumonia (COP). Treatment was initiated promptly with corticosteroids. Further HRCTs were done to assess the patient’s progress, and revealed a general improvement of the peri bronchial distribution of lung infiltrates.

In June 2021, she was diagnosed with acute diverticulitis with localized perforation and was managed conservatively. In October 2021, she contracted COVID-19. She recovered well but presented in December 2021 with a 3-week history of increasing breathlessness, symmetrical arm weakness and rash over her lower limbs. A creatine kinase (CK) was sent which came back with a high value of 3907 IU/L. She was then referred to the rheumatology team. Further investigations revealed a cytoplasmic ANA stain but negative myositis antibody profile, an increase in Troponin T, and pericardial effusion. MRI of the upper limbs showed acute proximal upper limb myositis, worse on the left. Subsequent muscle biopsy showed a necrotizing myopathy. The rash on her calves was initially described as a livedo-like rash, though with skin breakdown, necrotic and ulcerated areas. A skin biopsy was reported as non-uremic calciphylaxis.

She was treated for myositis with corticosteroids and intravenous immunoglobulins (chosen due to concerns regarding infection given the diverticular perforation). Her leg ulcers were regularly dressed with light compression and treated with antibiotics. These measures improved her muscle strength, CK and troponin. Leg ulcers were slow to heal but did improve over a period of ten months.

Regarding her abdominal perforation, she had two surgeries but developed small bowel obstruction in December 2022. Despite surgical intervention, her condition deteriorated and, unfortunately, she passed away.

**Discussion:**

The co-occurrence of calciphylaxis and myositis poses several interesting questions for medical professionals. The pathogenesis of calciphylaxis in end-stage renal disease is thought to be related to abnormalities in calcium and phosphate metabolism. However, in myositis it is unclear. Chronic inflammation and tissue damage associated with myositis likely play a role in the initiation of the calcification process. Patients with myositis-associated calciphylaxis typically present with painful skin lesions which often start as small, purplish, or mottled patches that progress to necrotic ulcers. These lesions most commonly occur in the lower extremities but can also involve the abdomen, buttocks, or upper extremities. The ulcers are often non-healing and prone to secondary infection leading to a considerable risk of complications. The condition is associated with significant morbidity and mortality.

Calciphylaxis in the context of myositis poses diagnostic and treatment challenges given the rarity of the condition. Differentiating it from other causes of tissue necrosis and skin ulcerations requires careful evaluation of clinical history, laboratory tests, imaging, and skin biopsies. This case involved a multidisciplinary approach including dermatologists, rheumatologists, vascular, pain management and wound care specialists. Treatment strategies aim to address wound care, pain management, secondary infection control, controlling inflammation in myositis, optimising renal function and potentially interventions targeting calcium and phosphate metabolism.

Overall, calciphylaxis in myositis presents a significant clinical challenge that requires early recognition, prompt diagnosis, and a multidisciplinary treatment approach for managing this complex condition and improving patient outcomes. This condition requires further research to better understand the pathogenesis and outcomes with different treatments.

**Key learning points:**

The most important learning point in this case for us was the multidisciplinary team required approach to manage this rare condition. Collaboration among rheumatology, dermatology, vascular and wound care specialties helped us to optimise treatment strategies for this condition. Identifying and addressing the underlying causes of calciphylaxis is essential. In myositis, autoimmune processes should be managed appropriately. Proper wound care is crucial to prevent infection and promote wound healing. Wound care specialists can provide guidance on appropriate dressings and techniques to optimise wound healing. Calciphylaxis-associated pain can be severe and may need involvement of pain management team for monitoring and adjustment of medications. Calciphylaxis can be a rare skin manifestation of inflammatory myositis.

